# Supplementary figures and images for: CAMSAP2 organizes a γ-tubulin-independent microtubule nucleation centre through phase separation
Source: eLife. 2022 Jun 28;11:e77365. doi: 10.7554/eLife.77365 (PMC9239687; doi:10.7554/eLife.77365)

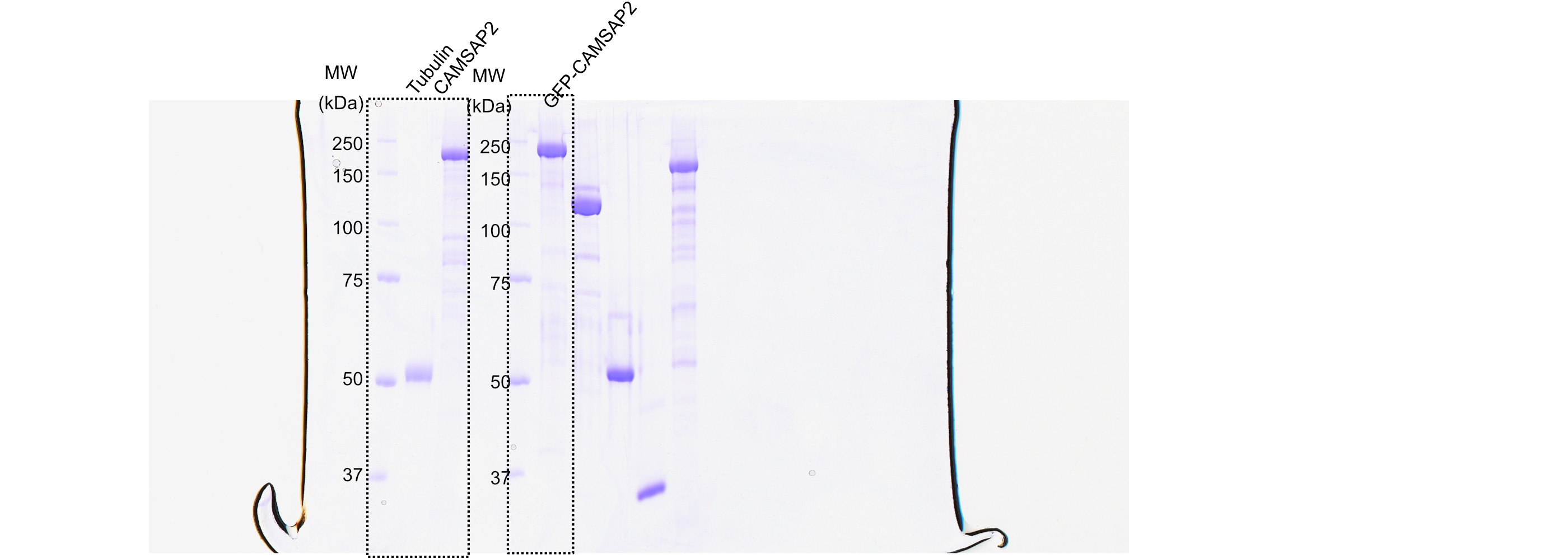

Supplement: Figure 1—source data 1. [file elife-77365-fig1-data1.jpg]

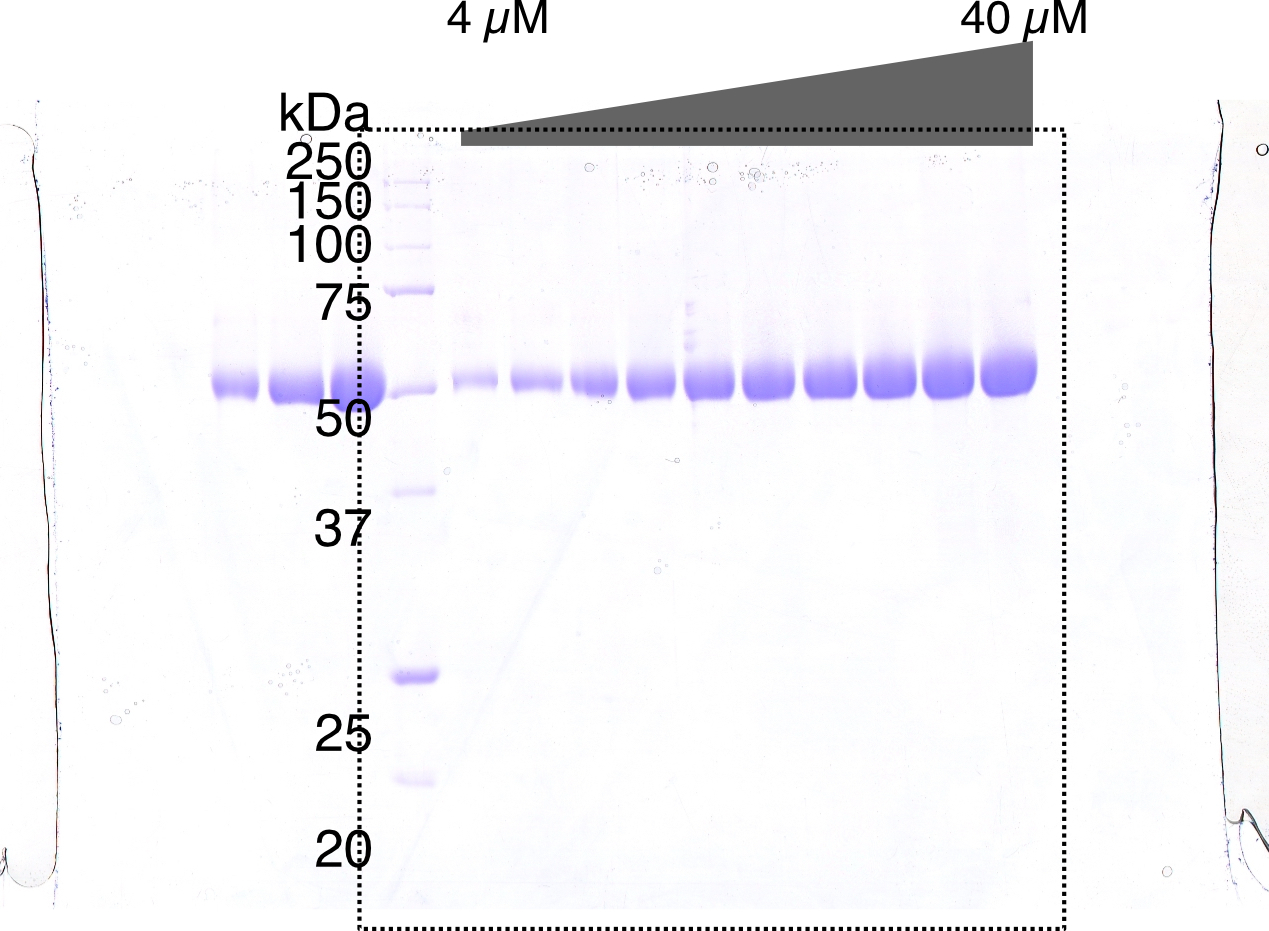

Supplement: Figure 2—source data 1. [file elife-77365-fig2-data1.jpg]

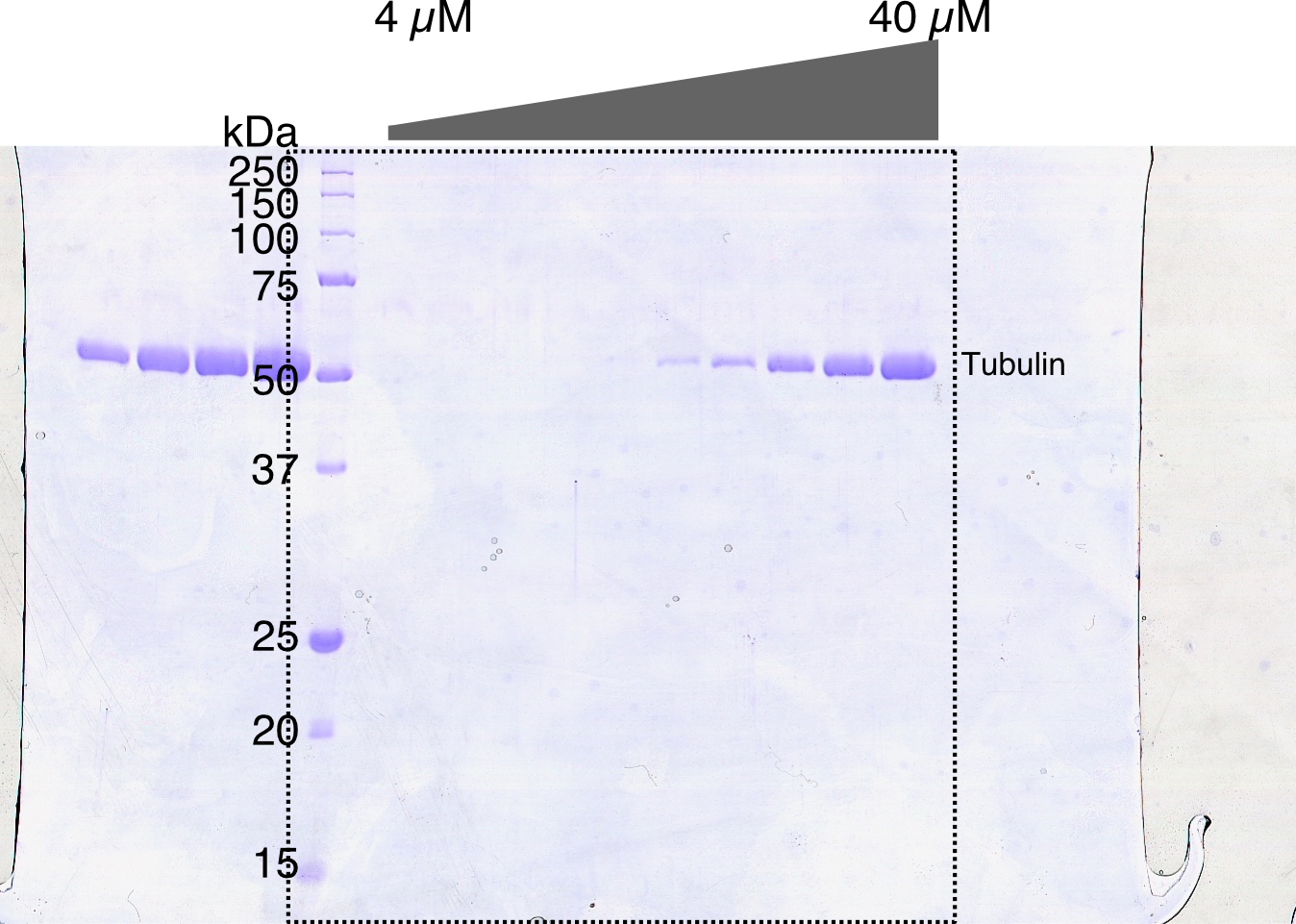

Supplement: Figure 2—source data 2. [file elife-77365-fig2-data2.jpg]

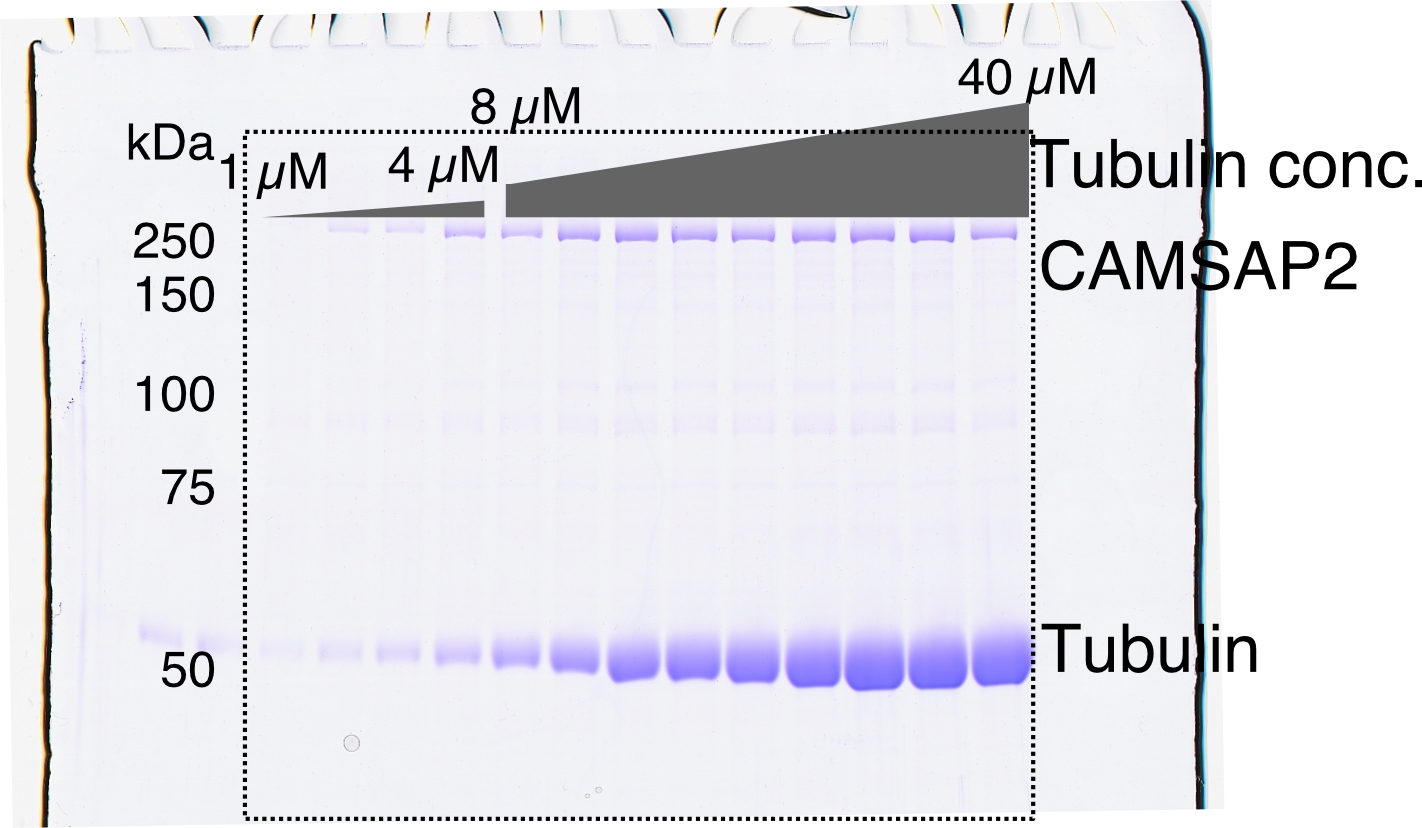

Supplement: Figure 2—source data 3. [file elife-77365-fig2-data3.jpg]

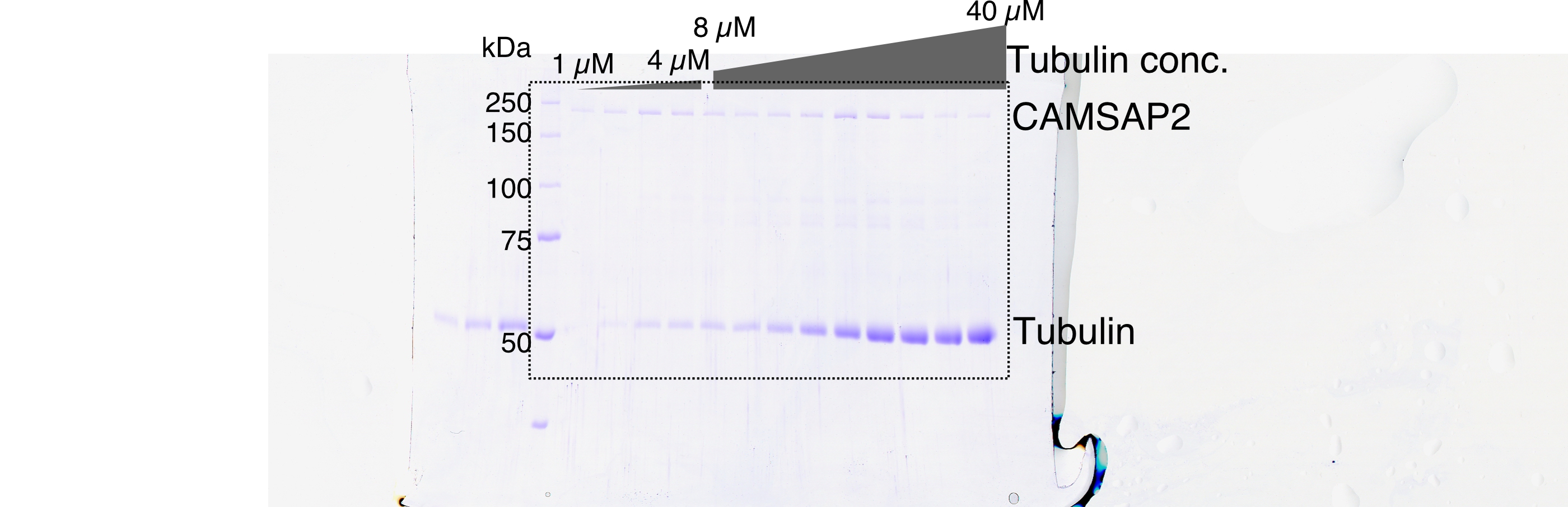

Supplement: Figure 2—source data 4. [file elife-77365-fig2-data4.jpg]

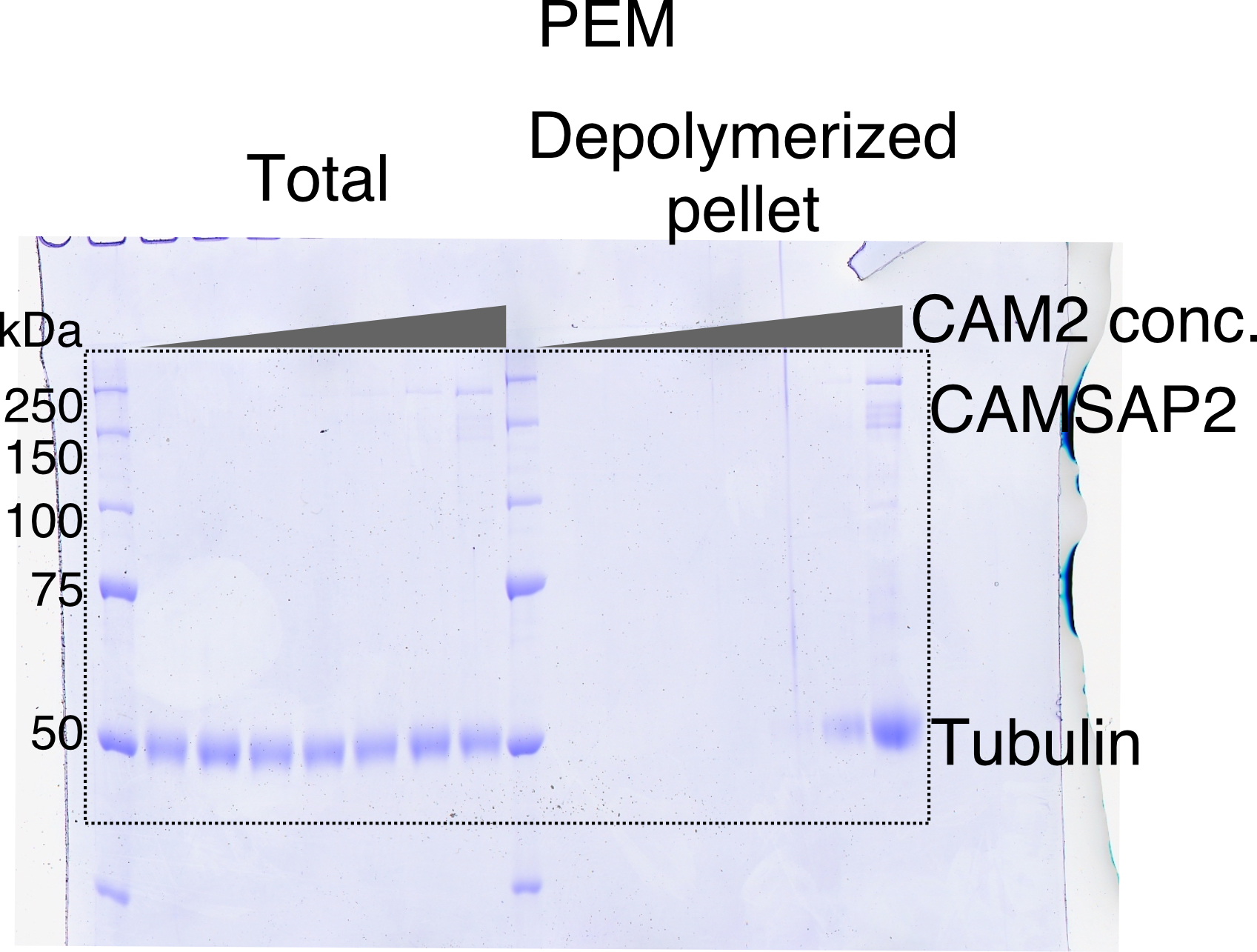

Supplement: Figure 2—source data 6. [file elife-77365-fig2-data6.jpg]

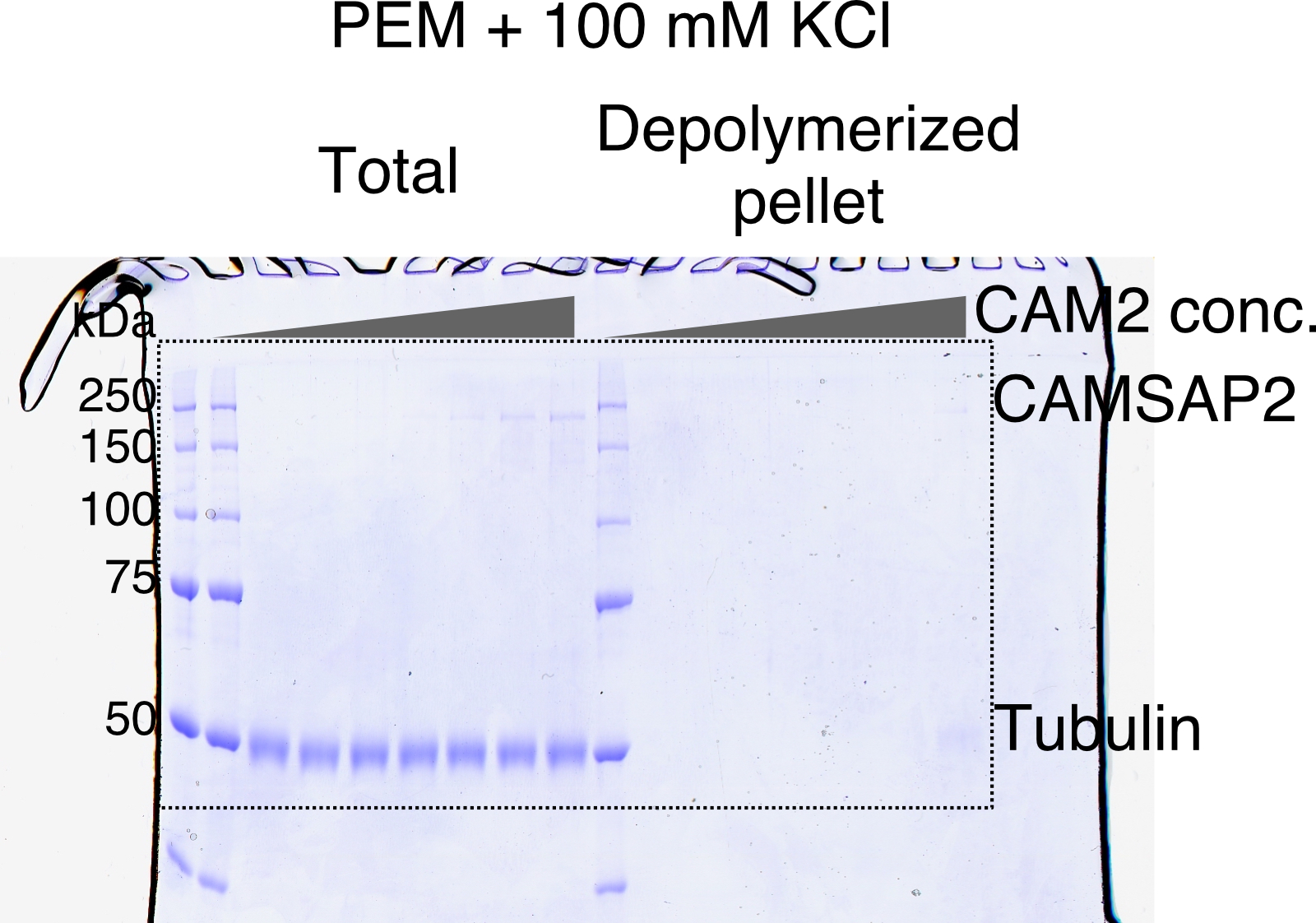

Supplement: Figure 2—source data 7. [file elife-77365-fig2-data7.jpg]

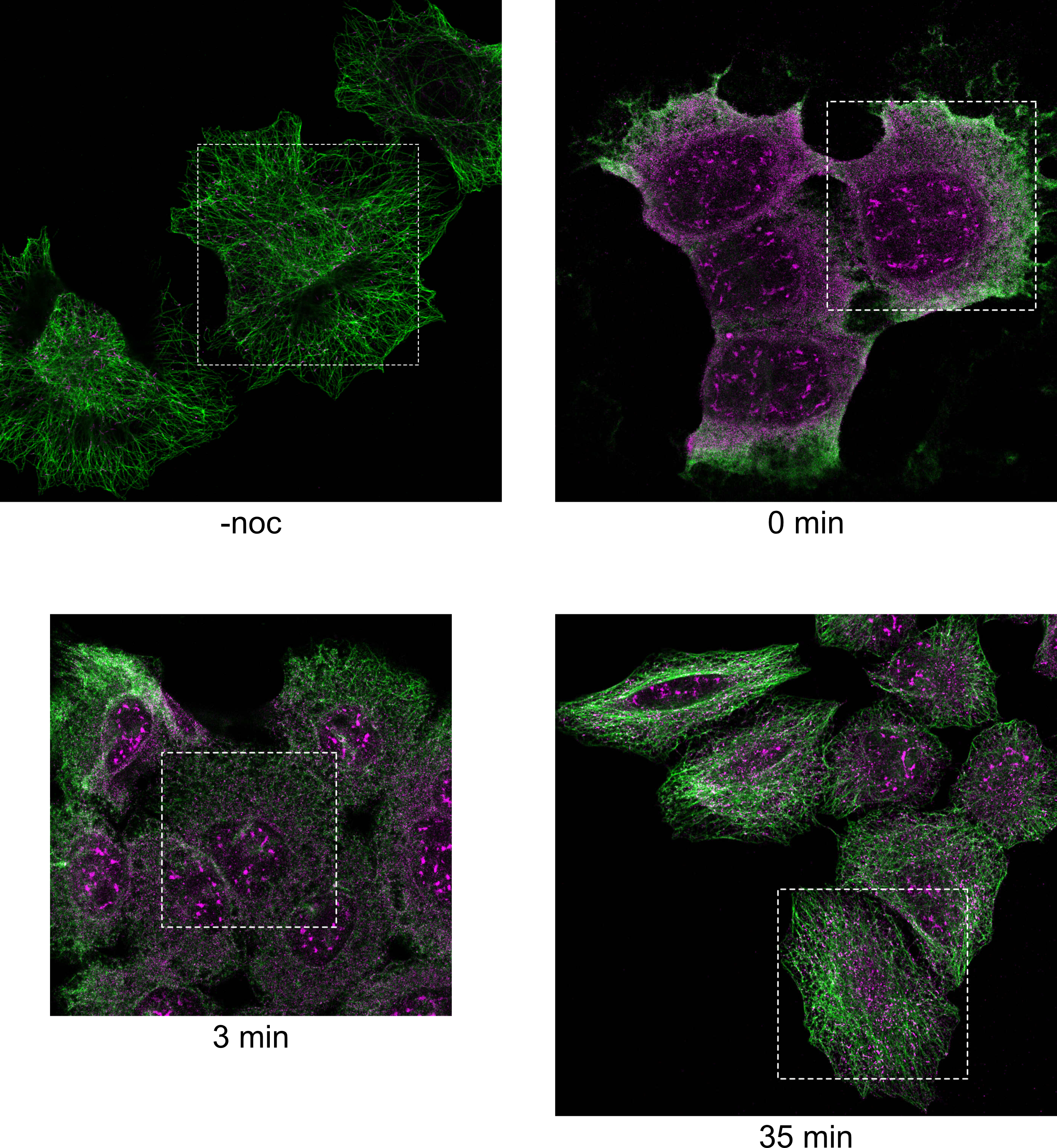

Supplement: Figure 4—figure supplement 1—source data 1. [file elife-77365-fig4-figsupp1-data1.jpg]

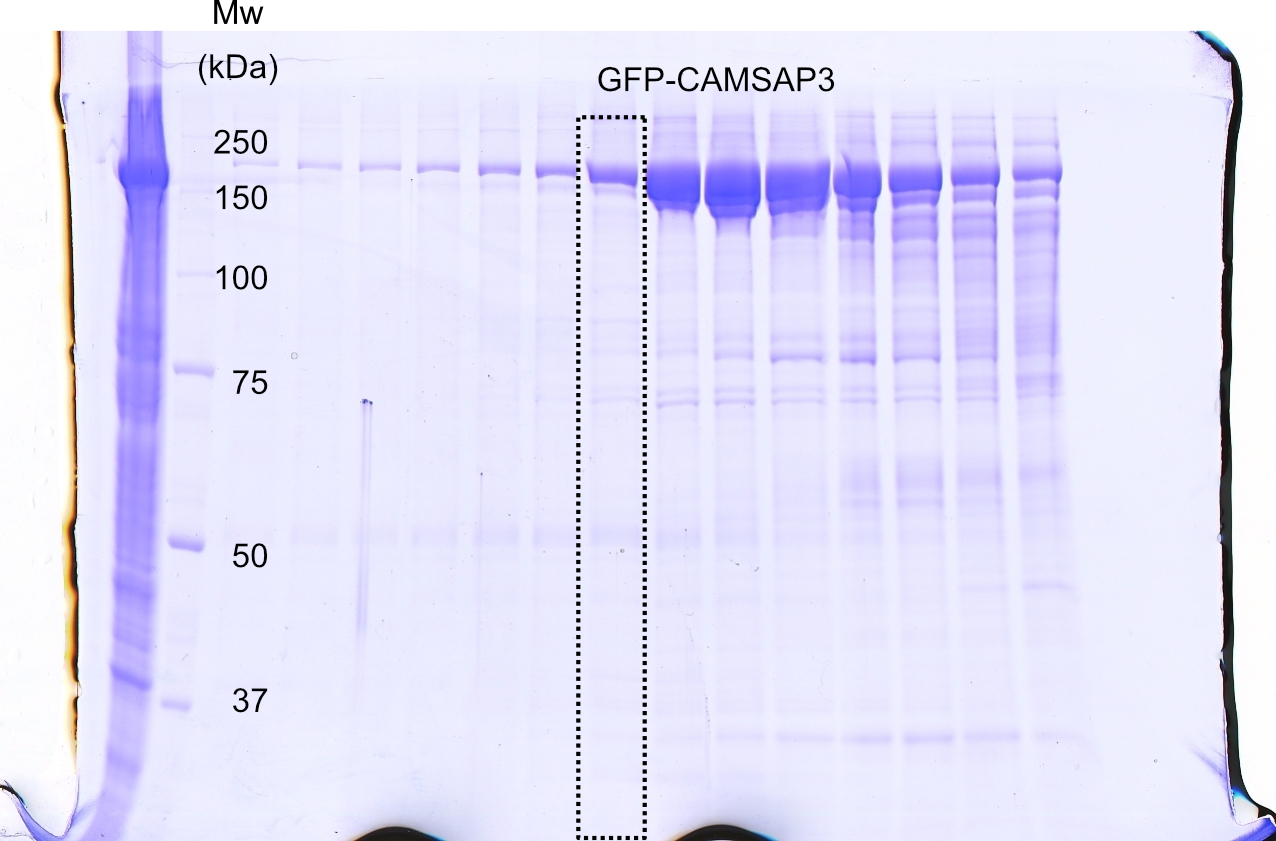

Supplement: Figure 5—figure supplement 1—source data 1. [file elife-77365-fig5-figsupp1-data1.jpg]

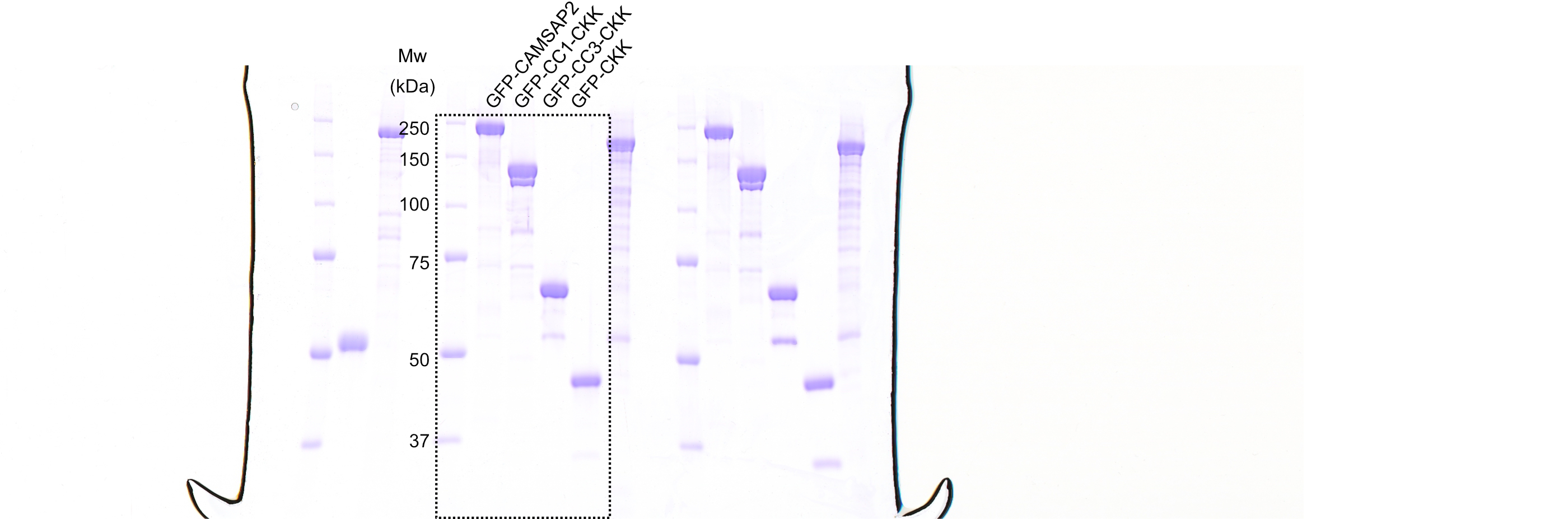

Supplement: Figure 6—figure supplement 1—source data 1. [file elife-77365-fig6-figsupp1-data1.jpg]

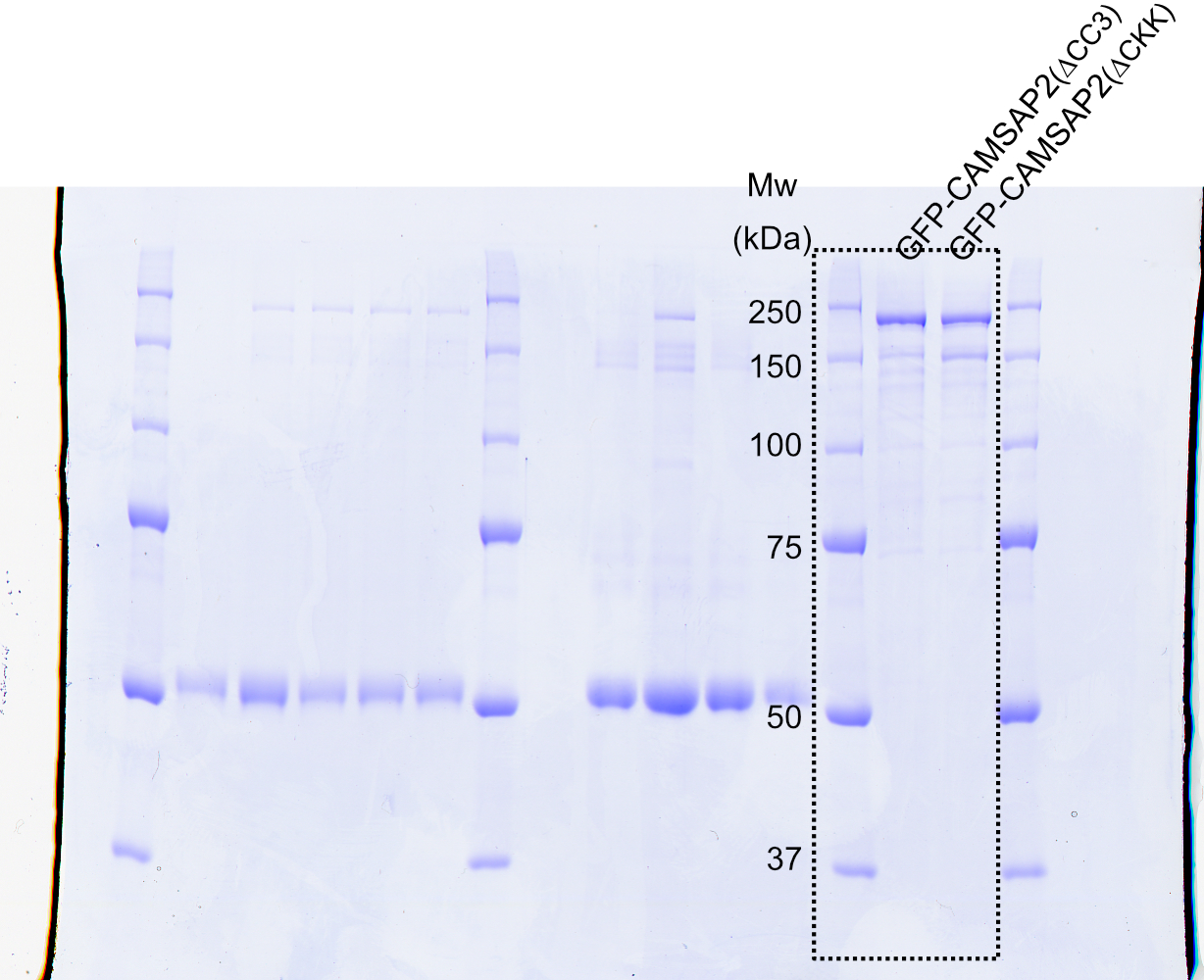

Supplement: Figure 6—figure supplement 1—source data 2. [file elife-77365-fig6-figsupp1-data2.jpg]
