# Supplementary material for: CAMSAP2 organizes a γ-tubulin-independent microtubule nucleation centre through phase separation
Source: eLife. 2022 Jun 28;11:e77365. doi: 10.7554/eLife.77365 (PMC9239687; doi:10.7554/eLife.77365)
Supplement: Figure 2—source data 5. [file elife-77365-fig2-data5.docx]

| Tubulin pelleting assay | | |  |  |  |
| --- | --- | --- | --- | --- | --- |
| Tubulin |  |  |  |  |  |
|  |  |  |  |  |  |
| Tub Conc | Gel1 | Gel2 | Gel3 | average | sd |
| 4 | 0 | 0 | 0 | 0.00 |  |
| 8 | 0.00 | 0.00 | 0.00 | 0.00 |  |
| 12 | 0.00 | 0.00 | 0.00 | 0.00 |  |
| 16 | 0.00 | 0.01 | 0.00 | 0.00 |  |
| 20 | 0.01 | 0.02 | 0.00 | 0.01 |  |
| 24 | 0.15 | 0.11 | 0.20 | 0.15 | 0.05 |
| 28 | 0.96 | 0.19 | 0.66 | 0.60 | 0.39 |
| 32 | 2.91 | 1.00 | 1.67 | 1.86 | 0.97 |
| 36 | 2.79 | 2.29 | 2.68 | 2.59 | 0.26 |
| 40 | 3.85 | 2.16 | 3.94 | 3.32 | 1.00 |
|  |  |  |  |  |  |
|  |  |  |  |  |  |
| No depoly |  |  |  |  |  |
| Tub Conc | gel1 | gel2 | gel3 | average | sd |
| 4 | 0.00 | 0.00 | 0.00 | 0.00 | 0 |
| 8 | 0.00 | 0.00 | 0.00 | 0.00 | 0.00 |
| 12 | 0.00 | 0.00 | 0.00 | 0.00 | 0.00 |
| 16 | 0.00 | 0.00 | 0.00 | 0.00 | 0.00 |
| 20 | 0.00 | 0.00 | 0.00 | 0.00 | 0.00 |
| 24 | 0.09 | 0.00 | 0.70 | 0.26 | 0.38 |
| 28 | 1.14 | 0.92 | 1.00 | 1.02 | 0.11 |
| 32 | 0.98 | 0.35 | 3.19 | 1.51 | 1.49 |
| 36 | 1.49 | 3.03 | 4.81 | 3.11 | 1.66 |
| 40 | 2.37 | 3.38 | 2.84 | 2.86 | 0.51 |
