# Supplementary material for: CAMSAP2 organizes a γ-tubulin-independent microtubule nucleation centre through phase separation
Source: eLife. 2022 Jun 28;11:e77365. doi: 10.7554/eLife.77365 (PMC9239687; doi:10.7554/eLife.77365)
Supplement: Figure 2—source data 8. [file elife-77365-fig2-data8.docx]

| Tubulin pelleting assay | | |  |  |  |  |
| --- | --- | --- | --- | --- | --- | --- |
|  |  |  |  |  |  |  |
| PEM |  |  |  | PEM + 100 mM KCl | |  |
| conc | average | sd |  | conc | average | sd |
| 10 | 0.00 |  |  | 10 | 0.00 |  |
| 25 | 0.00 |  |  | 25 | 0.00 |  |
| 50 | 0.00 |  |  | 50 | 0.00 |  |
| 100 | 0.00 |  |  | 100 | 0.00 |  |
| 250 | 0.05 | 0.03 |  | 250 | 0.00 | 0.00 |
| 500 | 0.70 | 0.14 |  | 500 | 0.03 | 0.00 |
| 1000 | 1.83 | 0.21 |  | 1000 | 0.14 | 0.10 |
