# Supplementary material for: CAMSAP2 organizes a γ-tubulin-independent microtubule nucleation centre through phase separation
Source: eLife. 2022 Jun 28;11:e77365. doi: 10.7554/eLife.77365 (PMC9239687; doi:10.7554/eLife.77365)
Supplement: Figure 3—source data 1. [file elife-77365-fig3-data1.docx]

| ama | BL_corr3[t] |  |
| --- | --- | --- |
| 0 | 0.234174 | 0.12993376 |
| 10 | 0.254875 | 0.1409114 |
| 20 | 0.29455 | 0.15160231 |
| 30 | 0.319714 | 0.16201396 |
| 40 | 0.351266 | 0.17215367 |
| 50 | 0.362037 | 0.18202853 |
| 60 | 0.42011 | 0.19164547 |
| 70 | 0.428427 | 0.2010112 |
| 80 | 0.459255 | 0.21013231 |
| 90 | 0.422315 | 0.21901518 |
| 100 | 0.411065 | 0.22766602 |
| 110 | 0.455009 | 0.23609091 |
| 120 | 0.485298 | 0.24429575 |
| 130 | 0.495348 | 0.25228627 |
| 140 | 0.396883 | 0.26006808 |
| 150 | 0.452529 | 0.26764663 |
| 160 | 0.444779 | 0.27502724 |
| 170 | 0.450373 | 0.28221506 |
| 180 | 0.449168 | 0.28921514 |
| 190 | 0.461363 | 0.29603237 |
| 200 | 0.466526 | 0.30267154 |
| 210 | 0.476651 | 0.3091373 |
| 220 | 0.484739 | 0.31543418 |
| 230 | 0.495899 | 0.32156658 |
| 240 | 0.506909 | 0.3275388 |
| 250 | 0.506742 | 0.33335503 |
| 260 | 0.563949 | 0.33901934 |
| 270 | 0.557764 | 0.3445357 |
| 280 | 0.62403 | 0.34990797 |
| 290 | 0.515791 | 0.35513992 |
| 300 | 0.500947 | 0.36023522 |
| 310 | 0.565892 | 0.36519742 |
| 320 | 0.518549 | 0.37003002 |
| 330 | 0.502151 | 0.37473638 |
| 340 | 0.501846 | 0.37931982 |
| 350 | 0.550322 | 0.38378354 |
| 360 | 0.523668 | 0.38813067 |
| 370 | 0.550271 | 0.39236425 |
| 380 | 0.543544 | 0.39648725 |
| 390 | 0.60796 | 0.40050256 |
| 400 | 0.604523 | 0.40441299 |
| 410 | 0.568764 | 0.40822128 |
| 420 | 0.601683 | 0.41193009 |
| 430 | 0.665204 | 0.41554204 |
| 440 | 0.578821 | 0.41905964 |
| 450 | 0.59102 | 0.42248536 |
| 460 | 0.597269 | 0.4258216 |
| 470 | 0.601259 | 0.42907071 |
| 480 | 0.622529 | 0.43223494 |
| 490 | 0.68697 | 0.43531653 |
| 500 | 0.628646 | 0.43831762 |
| 510 | 0.647069 | 0.44124033 |
| 520 | 0.626723 | 0.44408669 |
| 530 | 0.640605 | 0.44685871 |
| 540 | 0.734297 | 0.44955833 |
| 550 | 0.570123 | 0.45218743 |
| 560 | 0.618995 | 0.45474786 |
| 570 | 0.696203 | 0.45724141 |
| 580 | 0.618973 | 0.45966983 |
| 590 | 0.587605 | 0.46203483 |
| 600 | 0.607284 | 0.46433804 |
| 610 | 0.672337 | 0.4665811 |
| 620 | 0.589673 | 0.46876557 |
| 630 | 0.615971 | 0.47089298 |
| 640 | 0.705903 | 0.47296483 |
| 650 | 0.701707 | 0.47498256 |
| 660 | 0.654095 | 0.47694758 |
| 670 | 0.650611 | 0.47886128 |
| 680 | 0.670312 | 0.48072499 |
| 690 | 0.642337 | 0.48254003 |
| 700 | 0.581558 | 0.48430765 |
| 710 | 0.660159 | 0.48602911 |
| 720 | 0.595249 | 0.4877056 |
| 730 | 0.610929 | 0.4893383 |
| 740 | 0.65142 | 0.49092836 |
| 750 | 0.694768 | 0.49247688 |
| 760 | 0.652626 | 0.49398496 |
| 770 | 0.654884 | 0.49545365 |
| 780 | 0.639054 | 0.49688397 |
| 790 | 0.699347 | 0.49827694 |
| 800 | 0.655168 | 0.49963352 |
| 810 | 0.696003 | 0.50095466 |
| 820 | 0.667724 | 0.5022413 |
| 830 | 0.713476 | 0.50349433 |
| 840 | 0.729236 | 0.50471464 |
| 850 | 0.734173 | 0.50590307 |
| 860 | 0.694186 | 0.50706045 |
| 870 | 0.731234 | 0.50818761 |
| 880 | 0.695772 | 0.50928533 |
| 890 | 0.685297 | 0.51035437 |
| 900 | 0.639587 | 0.51139549 |
| 910 | 0.700802 | 0.51240942 |
| 920 | 0.668937 | 0.51339686 |
| 930 | 0.622999 | 0.51435851 |
| 940 | 0.709004 | 0.51529504 |
| 950 | 0.694803 | 0.51620711 |
| 960 | 0.653265 | 0.51709536 |
| 970 | 0.696176 | 0.51796041 |
| 980 | 0.663803 | 0.51880286 |
| 990 | 0.637008 | 0.51962331 |
| 1000 | 0.628301 | 0.52042232 |
| 1010 | 0.627485 | 0.52120047 |
| 1020 | 0.748902 | 0.52195829 |
| 1030 | 0.661123 | 0.52269632 |
| 1040 | 0.716178 | 0.52341507 |
| 1050 | 0.765377 | 0.52411505 |
| 1060 | 0.724396 | 0.52479674 |
| 1070 | 0.703819 | 0.52546063 |
| 1080 | 0.693354 | 0.52610718 |
| 1090 | 0.757636 | 0.52673684 |
| 1100 | 0.780074 | 0.52735005 |
| 1110 | 0.755598 | 0.52794725 |
| 1120 | 0.76328 | 0.52852884 |
| 1130 | 0.788605 | 0.52909525 |
