# Supplementary material for: CAMSAP2 organizes a γ-tubulin-independent microtubule nucleation centre through phase separation
Source: eLife. 2022 Jun 28;11:e77365. doi: 10.7554/eLife.77365 (PMC9239687; doi:10.7554/eLife.77365)
Supplement: Figure 3—figure supplement 1—source data 1. [file elife-77365-fig3-figsupp1-data1.pdf]

Repeat1

ama	BL_corr3[t]	
0	0.25222	0.13414975
10	0.291598	0.14558705
20	0.287226	0.15670174
30	0.342844	0.16750291
40	0.407902	0.17799941
50	0.413999	0.18819982
60	0.422893	0.19811251
70	0.47262	0.20774558
80	0.488638	0.21710693
90	0.452117	0.22620422
100	0.454716	0.23504489
110	0.428047	0.2436362
120	0.499718	0.25198516
130	0.502194	0.26009862
140	0.421089	0.26798322
150	0.468808	0.27564541
160	0.475823	0.28309148
170	0.472363	0.2903275
180	0.485723	0.29735942
190	0.477556	0.30419298
200	0.480154	0.31083379
210	0.542533	0.31728728
220	0.51643	0.32355873
230	0.524221	0.32965327
240	0.552451	0.33557591
250	0.534093	0.34133148
260	0.539068	0.3469247
270	0.611688	0.35236015
280	0.575086	0.35764228
290	0.554343	0.36277541
300	0.553313	0.36776375
310	0.535171	0.37261139
320	0.52977	0.37732228
330	0.581457	0.38190029
340	0.55802	0.38634916
350	0.576124	0.39067255
360	0.594937	0.39487398
370	0.547992	0.3989569
380	0.562185	0.40292465
390	0.558171	0.40678048
400	0.624391	0.41052755
410	0.610089	0.41416892
420	0.644697	0.41770758
430	0.626747	0.42114642
440	0.615981	0.42448826
450	0.650033	0.42773583
460	0.61895	0.4308918
470	0.672048	0.43395875
480	0.673994	0.43693918
490	0.63169	0.43983555
500	0.686609	0.44265021
510	0.664379	0.44538548
520	0.65954	0.4480436
530	0.687855	0.45062673
540	0.656843	0.45313701
550	0.628094	0.45557647
560	0.647758	0.45794712
570	0.676834	0.46025091
580	0.697474	0.4624897
590	0.656675	0.46466535
600	0.673314	0.46677963
610	0.66167	0.46883427
620	0.636226	0.47083095
630	0.646208	0.47277131
640	0.660423	0.47465694
650	0.6621	0.47648938
660	0.650111	0.47827013
670	0.594463	0.48000066

Repeat2

ama	BL_corr3[t]	
0	0.25167	0.10825171
10	0.289514	0.11253529
20	0.288403	0.11676422
30	0.294187	0.12093921
40	0.377704	0.12506094
50	0.42254	0.1291301
60	0.401527	0.13314735
70	0.414962	0.13711335
80	0.447249	0.14102877
90	0.359115	0.14489424
100	0.352501	0.1487104
110	0.369204	0.15247789
120	0.378826	0.15619732
130	0.40814	0.1598693
140	0.37169	0.16349444
150	0.374995	0.16707334
160	0.361235	0.17060659
170	0.342917	0.17409477
180	0.404858	0.17753845
190	0.361292	0.18093821
200	0.351745	0.1842946
210	0.39812	0.18760817
220	0.37853	0.19087948
230	0.388948	0.19410906
240	0.408805	0.19729744
250	0.40705	0.20044515
260	0.40472	0.20355271
270	0.42604	0.20662063
280	0.394622	0.20964941
290	0.41613	0.21263956
300	0.419403	0.21559157
310	0.416722	0.21850592
320	0.455674	0.2213831
330	0.426304	0.22422358
340	0.413817	0.22702782
350	0.467866	0.22979629
360	0.45773	0.23252945
370	0.45267	0.23522774
380	0.453587	0.23789162
390	0.440903	0.24052151
400	0.443111	0.24311786
410	0.481149	0.24568109
420	0.445319	0.24821162
430	0.46597	0.25070987
440	0.469246	0.25317626
450	0.496869	0.25561118
460	0.493647	0.25801505
470	0.506457	0.26038825
480	0.488838	0.26273118
490	0.493869	0.26504422
500	0.512441	0.26732776
510	0.519897	0.26958216
520	0.538191	0.27180782
530	0.539147	0.27400508
540	0.463262	0.27617431
550	0.489506	0.27831587
560	0.497398	0.28043012
570	0.48953	0.28251739
580	0.466444	0.28457804
590	0.478615	0.28661241
600	0.49562	0.28862082
610	0.5001	0.29060362
620	0.494187	0.29256112
630	0.483288	0.29449365
640	0.492864	0.29640154
650	0.516821	0.29828508
660	0.507417	0.3001446
670	0.505333	0.3019804

Repeat3

ama	BL_corr3[t]	
0	0.207129	0.07793074
10	0.257711	0.08207288
20	0.285447	0.08615126
30	0.270008	0.09016685
40	0.337378	0.09412062
50	0.349369	0.09801352
60	0.360759	0.1018465
70	0.378487	0.10562048
80	0.402454	0.10933635
90	0.303349	0.11299502
100	0.345002	0.11659737
110	0.364649	0.12014426
120	0.375369	0.12363656
130	0.350138	0.12707509
140	0.332813	0.13046068
150	0.335166	0.13379416
160	0.319096	0.13707632
170	0.345017	0.14030796
180	0.348812	0.14348984
190	0.369434	0.14662275
200	0.341641	0.14970742
210	0.371008	0.15274461
220	0.352936	0.15573504
230	0.342028	0.15867944
240	0.341895	0.16157851
250	0.360793	0.16443295
260	0.376504	0.16724345
270	0.362212	0.17001068
280	0.388219	0.17273532
290	0.352485	0.17541801
300	0.364362	0.1780594
310	0.391535	0.18066013
320	0.374613	0.18322083
330	0.406899	0.1857421
340	0.410424	0.18822456
350	0.383888	0.1906688
360	0.383663	0.19307542
370	0.379233	0.19544499
380	0.394315	0.19777808
390	0.399132	0.20007526
400	0.439689	0.20233707
410	0.40746	0.20456406
420	0.428638	0.20675677
430	0.411096	0.20891573
440	0.398411	0.21104145
450	0.41106	0.21313444
460	0.40595	0.21519522
470	0.46051	0.21722427
480	0.457392	0.21922208
490	0.455971	0.22118914
500	0.445178	0.22312592
510	0.479247	0.22503288
520	0.438273	0.22691049
530	0.502517	0.22875919
540	0.466167	0.23057944
550	0.445651	0.23237166
560	0.422581	0.23413629
570	0.478673	0.23587376
580	0.440314	0.23758448
590	0.433579	0.23926886
600	0.433575	0.24092732
610	0.458733	0.24256024
620	0.473445	0.24416803
630	0.425407	0.24575106
640	0.475681	0.24730973
650	0.499073	0.2488444
660	0.46217	0.25035544
670	0.443474	0.25184323

680	0.694702	0.48168236
690	0.675437	0.48331663
700	0.606392	0.4849048
710	0.619586	0.48644818
720	0.646212	0.48794802
730	0.595354	0.48940555
740	0.729006	0.49082197
750	0.692949	0.49219843
760	0.630854	0.49353607
770	0.668579	0.49483598
780	0.724069	0.49609921
790	0.744786	0.49732682
800	0.735055	0.4985198
810	0.741426	0.49967913
820	0.745637	0.50080575
830	0.72167	0.5019006
840	0.710457	0.50296456
850	0.771341	0.50399852
860	0.74197	0.5050033
870	0.683268	0.50597975
880	0.716465	0.50692865
890	0.6643	0.50785078
900	0.62858	0.50874691
910	0.730871	0.50961775
920	0.688433	0.51046404
930	0.67148	0.51128645
940	0.666843	0.51208566
950	0.666391	0.51286233
960	0.696158	0.51361709
970	0.690961	0.51435056
980	0.719682	0.51506334
990	0.715022	0.51575602
1000	0.664601	0.51642915
1010	0.711431	0.5170833
1020	0.702027	0.517719
1030	0.645487	0.51833677
1040	0.752977	0.51893711
1050	0.678997	0.51952051
1060	0.769	0.52008746
1070	0.68848	0.52063842
1080	0.792269	0.52117384
1090	0.73114	0.52169415
1100	0.766088	0.52219979
1110	0.749883	0.52269116
1120	0.763192	0.52316867
1130	0.806589	0.52363272

680	0.536533	0.30379278
690	0.505688	0.30558204
700	0.472003	0.30734848
710	0.528946	0.30909239
720	0.540441	0.31081405
730	0.536651	0.31251375
740	0.558469	0.31419177
750	0.552391	0.31584838
760	0.546654	0.31748386
770	0.550645	0.31909848
780	0.55555	0.32069251
790	0.538168	0.3222662
800	0.508809	0.32381981
810	0.525911	0.32535361
820	0.539989	0.32686785
830	0.579765	0.32836277
840	0.528253	0.32983861
850	0.530218	0.33129564
860	0.536242	0.33273408
870	0.51255	0.33415417
880	0.553119	0.33555614
890	0.53656	0.33694023
900	0.532104	0.33830667
910	0.482176	0.33965567
920	0.569296	0.34098747
930	0.531603	0.34230228
940	0.590112	0.34360032
950	0.56924	0.3448818
960	0.58594	0.34614693
970	0.558471	0.34739593
980	0.586062	0.34862899
990	0.537172	0.34984632
1000	0.539941	0.35104813
1010	0.560948	0.35223461
1020	0.573003	0.35340595
1030	0.59541	0.35456235
1040	0.553732	0.355704
1050	0.580921	0.35683108
1060	0.572427	0.35794379
1070	0.596832	0.35904231
1080	0.59748	0.36012681
1090	0.552673	0.36119748
1100	0.57684	0.36225449
1110	0.575417	0.36329802
1120	0.586614	0.36432824
1130	0.593359	0.36534531

680	0.452031	0.25330811
690	0.44513	0.25475044
700	0.490949	0.25617057
710	0.474332	0.25756883
720	0.503499	0.25894557
730	0.484021	0.26030112
740	0.477035	0.2616358
750	0.518926	0.26294993
760	0.475606	0.26424383
770	0.495042	0.26551781
780	0.482105	0.26677218
790	0.472225	0.26800724
800	0.479873	0.26922329
810	0.482267	0.27042062
820	0.508806	0.27159952
830	0.465825	0.27276026
840	0.480549	0.27390314
850	0.497513	0.27502843
860	0.487808	0.27613639
870	0.492915	0.27722729
880	0.458101	0.27830141
890	0.487469	0.27935898
900	0.460973	0.28040028
910	0.454524	0.28142554
920	0.476548	0.28243503
930	0.494368	0.28342897
940	0.461924	0.28440761
950	0.47041	0.28537119
960	0.516922	0.28631993
970	0.518412	0.28725407
980	0.464527	0.28817382
990	0.542516	0.28907942
1000	0.472563	0.28997108
1010	0.485632	0.29084901
1020	0.507116	0.29171343
1030	0.503148	0.29256453
1040	0.490477	0.29340254
1050	0.532565	0.29422765
1060	0.490073	0.29504005
1070	0.493507	0.29583995
1080	0.538097	0.29662753
1090	0.46314	0.29740299
1100	0.540105	0.29816651
1110	0.523481	0.29891828
1120	0.537761	0.29965847
1130	0.528817	0.30038727
