# Supplementary material for: CAMSAP2 organizes a γ-tubulin-independent microtubule nucleation centre through phase separation
Source: eLife. 2022 Jun 28;11:e77365. doi: 10.7554/eLife.77365 (PMC9239687; doi:10.7554/eLife.77365)
Supplement: Figure 6—source data 1. [file elife-77365-fig6-data1.pdf]

Full length	CC1-CKK	CC3-CKK
18747.47	25078.35	3085.581
83693.64	24699.64	1171.466
25730.87	9782.182	1894.434
32941.46	11822.18	875.7019
43029.78	10510.75	1735.778
16500.07	20646.86	2180.827
18583.46	320.58	1385.943
27607.13	17046.51	2088.197
8119.855	9339.264	2075.767
6079.744	15100.88	2923.762
11636.42	18209.59	4141.859
38094.9	13977.35	3868.955
12046.48	4216.028	1034.292
24077.54	7784.669	2188.433
31854.55	16018.8	1303.421
2738.278	11948.24	1819.892
12641.32	7537.796	1445.29
9015.745	14523.14	1036.055
21505.55	36508.18	3404.477
43428.2	16207.95	2667.782
34253.85	22105.07	1115.245
92647.37	19499.99	1573.576
10323.06	19890.04	4867.455
23269.27	8480.875	1893.079
16782.68	7260.752	5994.384
12664.76	25788.14	4692.805
16682.02	25332.63	2305.067
36075.28	19358.69	2122.498
7112.028	17844.92	2398.807
49214.36	20433.06	3387.289
61160.97	18434.42	4057.938
54015.41	16625.92	3179.191
24688.04	12622.49	3164.477
48677.63	15227.92	6628.158
55651.01	6344.795	3588.399
17872.23	16829.04	1470.087
20617.32	9362.043	3688.56
58221.02	7377.568	3076.46
25173.6	10237.65	1959.901
33808.84	6383.476	3268.929
42833.15	6269.091	3279.692
52130.02	9639.022	2044.259
29751.04	13847.82	2065.92
23618.11	28011.5	1388.226
49028.73	27262.16	1655.926
105990.4	21078.49	2020.212
95739.2	27671.42	2400.434

52888.94	12226.15	1873.222
27456.24	10296.88	1613.119
23745.78	12550.24	3967.444
107285.3	13825.2	3528.004
34865.98	15453.99	3111.948
17371.87	11023.26	3438.618
26190.98	48236.02	2250.386
265.538	31701.86	5957.429
32350.88	20437.41	3585.139
42337.47	11430.31	1950.847
30954.49	22291.38	2309.534
39230.26	14130.07	36.3
83635.41	27371.5	4283.409
30696.04	9829.004	2056.309
78623.54	8618.954	1228.239
33884.07		3002.454
83881.78	35748.46	5345.961
84715.57	12737.51	1950.416
97570.25	16844.33	1193.688
127302.4	285.194	2969.21
82766.65	10585.52	2631.178
118867.2	1098.994	1960.658
111096.9	22674.51	2647.098
52296.68	27490.07	4057.246
37293.71	384.54	4142.348
13829.63	35901.19	5755.974
20506.5	36396.59	3479.911
48869.14	48077.43	4267.061
14779.85	18506.86	2734.461
66116.35	12558.38	3472.533
26263.63	20872.65	3317.672
32671.71	25358.92	3098.589
44374.02	19323.16	4413.732
75472.91	22225.1	6998.969
26515.64	14522.66	5046.792
86694.88	22310.22	50.171
17706.88	30976.33	2097.574
25779.75	17134.35	3477.107
32365.38	20313.97	4101.97
63357.48	27578.15	2067.636
45713.22	25925.21	3124.685
14397.54	12368.88	2295.719
51409.92	17052.29	1846.195
94652.63	18037.48	2469.715
19325.18	37539.73	2379.03
112288.8	18297.55	1565.472
10914.95	20874.56	2866.168
83325.08	17026.81	4323.507

31113.74	35800.01	1761.805
25430.18	37970.2	4354.357
20106.67	20538.41	4059.84
67593.52	16881.08	3049.046
55352.28	17878.66	5146.481
