# Supplementary material for: CAMSAP2 organizes a γ-tubulin-independent microtubule nucleation centre through phase separation
Source: eLife. 2022 Jun 28;11:e77365. doi: 10.7554/eLife.77365 (PMC9239687; doi:10.7554/eLife.77365)
Supplement: Figure 7—source data 1. [file elife-77365-fig7-data1.pdf]

number of tubulin rings and that of microtubules

No. of rings

Tubulin conc.	Tub 10uM	Tub 10uM	Tub 10uM	Tub 10uM
CAMSAP CC1-CC3 conc.	1uM	1uM	1uM	1uM
Time (min)	On Ice	1	3	10
Ring No.	30	6	5	2
/0.75 um^2	20	6	6	2
	31	11	9	0
	20	10	3	0
	35	8	5	3
	19	6	7	3
	59	14	3	0
	33	21	6	0
Average	30.875	10.25	5.5	1.25
SD	13.0432	5.2030	2.0000	1.3887

No. of microtubules

Tubulin conc.	Tub 10uM	Tub 10uM	Tub 10uM	Tub 10uM
CAMSAP CC1-CC3 conc.	1uM	1uM	1uM	1uM
Time (min)	On Ice	1min	3min	10min
Microtubule No.	0	17	33	35
/0.75 um^2	0	16	26	34
	0	15	9	30
	0	13	26	21
	0	8	23	23
	0	5	28	21
	0	0	24	18
	0	6	25	25
Average	0	10	24.25	25.875
SD	0.0000	6.1412	6.8817	6.3794

Converted to /1 um^2

Time	Ring	MT	sd_ring	sd_MT
0	54.9	18.2	0.0	0.0
1	18.2	9.2	17.8	10.9
3	9.8	3.6	43.1	12.2
10	2.2	2.5	46.0	11.3
